# The Enigma of Heat Shock Proteins in Immune Tolerance

**DOI:** 10.3389/fimmu.2017.01599

**Published:** 2017-11-21

**Authors:** Willem van Eden, Manon A. A. Jansen, Irene Ludwig, Peter van Kooten, Ruurd van der Zee, Femke Broere

**Affiliations:** ^1^Department of Infectious Diseases and Immunology, Faculty of Veterinary Medicine (FVM), Utrecht University, Utrecht, Netherlands

**Keywords:** tolerance mechanisms, regulatory T cells, heat shock proteins, arthritis, rheumatoid, cell stress

## Abstract

The fundamental problem of autoimmune diseases is the failure of the immune system to downregulate its own potentially dangerous cells, which leads to destruction of tissue expressing the relevant autoantigens. Current immunosuppressive therapies offer relief but fail to restore the basic condition of self-tolerance. They do not induce long-term physiological regulation resulting in medication-free disease remissions. Heat shock proteins (HSPs) have shown to possess the capacity of inducing lasting protective immune responses in models of experimental autoimmune diseases. Especially mycobacterial HSP60 and HSP70 were shown to induce disease inhibitory IL-10-producing regulatory T cells in many different models. This in itself may seem enigmatic, since based on earlier studies, HSPs were also coined sometimes as pro-inflammatory damage-associated molecular patterns. First clinical trials with HSPs in rheumatoid arthritis and type I diabetes have also indicated their potential to restore tolerance in autoimmune diseases. Data obtained from the models have suggested three aspects of HSP as being critical for this tolerance promoting potential: 1. evolutionary conservation, 2. most frequent cytosolic/nuclear MHC class II natural ligand source, and 3. upregulation under (inflammatory) stress. The combination of these three aspects, which are each relatively unique for HSP, may provide an explanation for the enigmatic immune tolerance promoting potential of HSP.

## Introduction

Initial observations concerning the significance of heat shock proteins (HSP) for immune tolerance were obtained in the model of heat-killed mycobacteria induced adjuvant arthritis (AA) in Lewis rats. In this model, T cell clones, having only one singular T cell receptor (TCR), had been established by limiting dilutions following repeated re-stimulations with mycobacterial antigens. Upon *in vivo* transfer, these clones had the capacity to produce induction and suppression of the disease (1). These T cell lines had been raised from immunizations and repeated *in vitro* re-stimulations of collected splenocytes with crude heat-killed *Mycobacterium tuberculosis*. These T cell lines were found to respond with specificity to some mycobacterial protein fractions. And interestingly, these same mycobacterial protein fractions were also capable of inducing T cell responses in peripheral blood mononuclear cells obtained from RA patients (2). Since then it became of interest to define the exact nature of the antigens involved. The antigen recognized by T cell clone A2c, the clone with the capacity to protect against induction of AA, was obtained by molecular cloning of *M. tuberculosis*-derived genes (3). It was a 60-kDa protein which was found upon immunization to protect against AA. Based on sequence homologies with known HSPs, such as the 60-kDa GroEL of *Escherichia coli*, this protein was recognized as the mycobacterial HSP60. Subsequently, this molecule was found to protect in various experimentally induced animal models, including collagen and avridine arthritis, NOD diabetes, experimental allergic encephalomyelitis, some allergic disorders, and atherosclerosis [reviewed in Ref. (4)].

Further exploration of this recombinant mycobacterial HSP60 in the AA model revealed the presence of at least nine different T cell epitopes in this HSP60 recognized by T cells in Lewis rats (5). Of these T cell epitopes, the sequence at positions 256–265 was most conserved. When T cell lines were generated against all nine distinct epitopes, only the T cell line with specificity for this conserved epitope protected upon *in vivo* transfer against induction of AA. And immunizations with synthetic peptides spanning the nine different epitopes showed that only the 256–265 peptide protected against disease. These findings had suggested that the induction of T cell regulation in the AA model depended on the cross-recognition of host-tissue expressed HSP60 by the mycobacterial HSP60-specific T cells. In more general terms, T cell responses to conserved sequences of microbial HSPs seemed to become endowed with the capacity to restore tolerance and to act as regulatory T cells (Tregs). And above all, whichever the exact interpretation of these findings could be, experiments performed by various groups had indicated the capacity of microbial HSP, and besides HSP60 also other HSPs, to induce a disease suppressive T cell response.

## The Controversy Around HSP and Their Possible Damage-Associated Molecular Pattern (DAMP) Activities

Intracellular HSPs are upregulated in cells under stress. If, and if so how HSPs are exported out of the cell has remained enigmatic. HSPs have no signal sequence for transport over cell membranes. Nonetheless, the extracellular presence of HSPs has been documented in various experimental systems. The controversy arises when the extracellular soluble HSPs are said to act as pro-inflammatory molecules, the so-called DAMPs. Such DAMP activities are somewhat difficult to reconcile with the fact that intracellular HSPs and their MHC presented peptides were seen to have anti-inflammatory disease suppressive activities in experimental models of chronic inflammation and in first clinical trials (4, 6–9). Part of the demonstrated pro-inflammatory effects may have arisen from the fact that earlier work by many different groups was performed with recombinant mycobacterial HSPs produced in *E. coli*. Although attempts were made and reported to purify the recombinant protein, several published claims regarding the pro-inflammatory nature of HSPs may have been based on the activity of LPS and possibly other contaminants present in the proteins. In various instances, when pure HSP proteins were tested, no inflammatory activity was observed (10). This, in combination with the findings of the anti-inflammatory effects of HSP in experimental models, seems to indicate that HSPs are missing several of the qualities of the so-called true DAMPs. As we have argued before, HSP can be rather DAMPERs of the immune response instead of DAMPs (11, 12).

By their aggressive nature, true DAMPs necessarily are residing intracellularly. HSPs, however, are often reported to have activities as extracellular mediators. In addition to this, when DCs are cultured in the presence of purified HSP, the DCs are not activated. In the case of mycobacterial HSP70, it was shown that it inhibited DCs in their maturation from bone marrow-derived precursors, it induced production of IL-10 in DCs and the treated DC reduced T cell proliferation (13). In another experimental setup, mycobacterial HSP70 was also shown to modulate DCs and to produce DCs that upon *in vivo* transfer inhibited experimental arthritis in mice (14). All latter observations are difficult to reconcile with pro-inflammatory DAMP-like activities being a natural characteristic of HSPs.

## HSP-Directed Immune Responses Present in Patients’ Disease Remission

An extensive analysis of T cell responses to HSP60 was made in patients with juvenile idiopathic arthritis (JIA) (15–17). JIA is a heterogeneous disease with subtypes. A major subtype is self-limiting, known as persistent oligoarticular JIA, in which a maximum of four joints is affected. This self-limiting nature of JIA is regarded to result from adequate immune regulation, through which the immune response has managed to restore tolerance for self. Although self-limiting, OA-JIA often causes permanent joint damage with lifelong disability. On the other hand, polyarticular JIA, with more than four joints affected in the first half year of the disease must result from a failure to restore tolerance. Oligoarticular forms of arthritis have shown to feature T cell responses to HSP60, whereas polyarticular JIA has not or at least much less (16). And in addition, a longitudinal follow-up of these OA-JIA patients showed that phases of disease remission were proceeded by phases of enhanced HSP60-specific T cell responses (17). These observations suggested that in patients with OA-JIA, HSP60-specific T cells contributed to regulation of disease. The production of IL-10 in peripheral blood mononuclear cells of the patients was fully in line with this possibility (18, 19).

Similar observations were made in patients with juvenile dermatomyositis (DM). Muscle biopsy samples from juvenile DM patients showed upregulation of Hsp60 and peripheral blood mononuclear cells showed proliferative responses in the presence of HSP60. Production of pro-inflammatory cytokines by muscle-derived T cells in response to Hsp60 was associated with a poor clinical prognosis, whereas human Hsp60-specific induction of IL-10 was followed by clinical remission (20).

In multiple sclerosis (MS), some studies have profiled antibody repertoires. In one of the studies, it appeared that relapsing-remitting MS was characterized by HSP70 autoantibodies. And this was not observed in both primary and secondary progressive MS. In other words, immune responses to HSP70 were associated with disease that exhibited the intrinsic capacity to control, to some extent, inflammation. In this study, antigen microarrays defined unique serum immune signatures linked to different stages and pathological processes in MS. In this case, immune responses to HSPs seemed associated with remitting forms of the disease (21).

## Induction of Tregs by HSP70 in a Mouse Arthritis Model and in Humans

In the model of proteoglycan-induced arthritis (PGIA) in BALB/c mice, we have performed epitope mapping of mycobacterial HSP70. Similar to what we had done earlier for HSP60 (5), we now identified a very conserved epitope which had close sequence homologies with multiple members of the mammalian HSP70 family of molecules. The peptide based on this mycobacterial HSP70 was named HSP70-B29. Recently, we showed that the HSP70-B29 peptide induced Tregs, a CD4+ T cell population with the intrinsic capacity to control inflammation. These Tregs are CD4+CD25+Foxp3+ and upon *in vivo* transfer the cells suppressed PGIA in mice (22). Furthermore, *in vivo* depletion of transferred Tregs, with a depleting antibody specific for the congenic CD90.1 marker, abrogated disease suppression. Transferred cells exhibited a stable phenotype and were found in joints and draining lymph nodes up to 2 months after transfer. In humans, B29 was a promiscuous binder for all major HLA-II molecules, HLADRB1* 04:01 in particular. Also in humans, B29-specific Tregs were detected (23). And importantly, these B29-specific T cells were shown to cross-recognize the mammalian HSP70 homologs. Initial experiments with human B29-pulsed tolerizing dendritic cells showed the ability to activate B29-specific T cells and induce a regulatory phenotype in these human T cells, as based on the expression of CD49b, LAG-3, and GITR (Nicolic and Roep, unpublished).

## Mechanisms through Which HSPs may Induce Tolerance Mediating Tregs

There are three possible mechanistic explanations that may act in synergy, for a tolerance promoting anti-inflammatory effect of HSPs. It is along these possibilities that further mechanistic research could be undertaken.

### HSPs Are Evolutionary Conserved

As mentioned earlier, evolutionary conservation of microbial HSPs has led to antigenic similarities with their mammalian self-homologs present in the host. Despite this, HSPs are quite immunogenic. The microbial molecule HSP60, for example, was known as the “common antigen of gram negatives” already before its molecular definition (24). In addition to this, T cells with specificity for the conserved parts of the molecules can be easily detected (5, 25). Therefore, in principle, the exposure to microbiota-associated HSP in the tolerizing gut mucosa (26–28) or exposure to commensal microbe-associated HSP in the skin (29) may trigger HSP-specific Tregs with focus on the relatively conserved—repeatedly encountered—parts of the molecules. When microbiota-associated bacteria are being sampled, such as in the case of the gut by macrophages or dendritic cells with their protrusions through the epithelial layer, and become transported to the mesenchymal lymph nodes, the intracellular presence will cause stress in these bacteria, leading to a further upregulation of bacterial HSP. By such means, the immune system will be familiar with the safe presence of microbiota-derived HSP and may well utilize this trustworthy set of antigens for maintaining mucosal tolerance at the level of T cells with respect to this safe set of microbial antigens. Various studies have documented the nature of the TCRs of gut Tregs. Based on the relatively unique nature of colonic Treg TCRs, it was postulated that Tregs in the colon may have differentiated extra-thymically due to contact with bacterial or food antigens (26). On the other hand, others have emphasized the presence of shared TCRs between thymic Tregs and colonic Tregs, suggesting the thymic selection on the basis of self-recognition and the expansion in the periphery through “recognition of cross-reactive microbial antigens in the intestines” (27, 28). Whichever scenario will be the dominant one, there is definite evidence for cognate interactions between gut Tregs and microbiota-associated antigens (30). By their nature, HSP may well constitute a set of microbiota-associated antigens that may dominate in this respect and that drive tolerance promoting Treg.

### HSPs Are the Most Frequent Cytosolic/Nuclear MHC Class II Natural Ligand Source

MHC molecules that reach the cell surface are conformationally dependent on the presence of peptides in their peptide-binding grooves. This is the case for MHC class I molecules and also for the MHC class II molecules that interact with our CD4+ Tregs. MHC elution studies have shown that HSPs are among the most frequent cytosolic/nuclear MHC class II natural ligand sources (22). In the supplementary data set of the paper of Paludan et al. (31), the more relevant HSP70 family members are listed, next to GAPDH, with the MHC II molecules they were eluted from. Therefore, even in absence of inflammation, HSPs form a major part of the normal MHCII ligandome. When presented by tolerizing DC in the tissues, HSP-specific Tregs may become induced, contributing to the “tolerance promoting” default setting of the healthy immune system.

Also for the thymus, MHC elution studies have shown the presence of stress-associated molecule fragments in the MHC–peptide matrix. In this case, stress-associated molecule fragments were retrieved more from MHCII molecules than from MHCI molecules, and this especially in the DC depleted, and therefore positively selecting, thymocytes (32). Interestingly enough, also a fragment of HSP70 was eluted in this manner which contained our HSP70-B29 peptide. Another study has presented the immuno-phenotyping of HSP-expressing cells in fetal and adult thymus (33). There was shown to exist the complete concordance of Lu5 (pancytokeratin), a marker for thymic epithelial cells, and HSP70. In fact, a strong expression of HSP70 (and HSP27) was detected in medullary and cortical thymic epithelial cells. It was furthermore shown that thymic DCs and macrophages (CD68+ cells) were negative for HSP70 expression. Altogether, whereas the negatively selecting bone marrow-derived cells were negative for HSP70, the positive selecting thymocytes were featuring a strong expression.

From this, it can be concluded that HSPs are well positioned to contribute to, thymus dependent, central tolerance, by positively selecting HSP-specific Tregs. In the periphery, the HSP ligands as bound in the MHCII peptide-binding clefts may furthermore assist to maintain such Tregs and to act as targets for the regulatory activity of the centrally selected Tregs. And in addition to this, also peripherally induced Tregs (Tr1?) may target such HSP ligands in the MHCII-binding clefts.

### HSPs Are Upregulated in Tissues under (Inflammatory) Stress

Tissue stress resulting from inflammation ensures upregulated levels of intracellular HSPs (34). In this manner, upregulated HSP presented by MHC may act as a functional biomarker of inflammation. This is especially the case for HSP70, as HSP70 is as a key regulator, molecularly involved with the process of chaperone-mediated autophagy (CMA). Dengjel et al. have analyzed the MHCII ligandome obtained from nutrient-deprived HLA-DR4+ human B cells (35). The stress caused by nutrient deprivation had led to autophagy, which influenced the loading of the MHCII compartments of the cell. Possibly through the mechanism of CMA, a chaperone-dependent targeting of cytosolic proteins to lysosomes, a preferential loading of MHCII with HSP70 fragments takes place. In the cleft of the HLA-DR4 molecules, also our HSP70-B29 was present in this case. Given the known association of HLA-DR4 with RA, this finding is of interest. Apparently, also RA patients with disease predisposing HLA molecules have in principle the genetic capacity to present a proposed disease protective peptide to their T cells.

The enhanced expression of mammalian HSP60 in synovial tissues of JIA patients has been reported by Boog et al. (36). A more complete study covering the upregulated expression of HSP in the inflamed synovium was reported by Schett et al. (37). An immunochemical analysis with HSP70-specific antibodies revealed strong staining in synovial fibroblasts and macrophages in the synovial tissues of patients with RA and not in those of patients with osteoarthritis. Induction of hsp70 expression and nuclear translocation of HSF1 in synovial cells was shown by immunofluorescence microscopy after incubation of synovial cells at raised temperature or incubation with TNF-α.

Under (sterile) inflammatory conditions, tolerizing DCs will have their MHC II more heavily loaded with HSP, which may enable them to herewith induce, expand, and activate HSP-specific Tregs at the sites of inflammation. Tregs may have evolved by the need to control inflammation and may depend for their function on a repertoire of TCRs that enables the recognition of HSP, in line with the abundant presence of HSP in the T cell-selecting thymus (32). Together with IL-10 as a regulatory cytokine also produced by cells under stress (38), it seems that cell-stress and control of inflammation are naturally connected.

Also by other means, HSPs may contribute to the inhibition of the inflammatory process. In various studies and interestingly also by various mechanisms, intracellular HSP70 was found to inhibit the inflammatory stimuli-dependent activation of the pro-inflammatory NF-κB signaling pathway (39, 40). Moreover, extracellular HSP70 was shown to have anti-inflammatory effects through inhibition of MAPKs and NF-κB signaling pathways leading to a downregulated production of IL-6, IL-8, and MCP-1 upon TNF-α stimulation of synoviocytes obtained from RA patients (41).

## Conclusion

Therefore, cell stress-associated intracellular HSPs have a tolerizing effect through T cells in combination with control of inflammatory mediator production. For these reasons, cell stress and the consequential expression of HSPs can be seen as a central element in the control of inflammation. As said, the “enigmatic” HSPs are no DAMPs, but rather tolerance promoting DAMPERs (11).

## Author Contributions

WE wrote the paper. The other authors edited the paper.

## Conflict of Interest Statement

WE has shares in Trajectum Pharma, a SME that develops HSP peptides for therapy. All other authors declare that the research was conducted in the absence of any commercial or financial relationships that could be construed as a potential conflict of interest. The reviewer LH and handling editor declared their shared affiliation.
